# A Regime of Health

**Published:** 1886-08

**Authors:** 


### *A REGIME OF HEALTH.*

### As Given 250 Years Ago by Lord Bacon.

There is a wisdom in this beyond the rules of phisic; a man's own observation, what he finds good of, and what he finds hurt of, is the best phisic to preserve health, but it is a safer conclusion to say, "this agreeth with me, therefore I will continue it: I find no offense of this, therefore I may use it;" for strength of nature in youth passes over many excesses, which are owing a man till his age; discern of the coming on of years, and think not to do the same things still: beware of any sudden change in any great point of diet: and if necessary enforce it, fit the rest to it: to be free-minded, and cheerfully disposed, at hours of meat, and of sleep, and of exercise, is the best precept of long lasting: if you fly phisic in health altogether, it will be too strong for your body when you shall need it: if you make it too familiar, it will work no extraordinary effect when sickness cometh: despise no new accident in the body, but ask opinion of it: in sickness principally respect health, and in health, action; for those that put their bodies to endure in health, may in most sicknesses, which are not very sharp, be cured only with diet and good tending: Physicians are some of them so pleasing to the humors of the patient, that they press not the true cure of the disease, and some others so regular in proceeding according to art for the disease, as they respect not sufficiently the condition of the patient: take one of a middle temper, and forget not to call as well the best acquainted with your body, as the best reputed of for his faculty.

